# Development, validation and reliability of scales and items for heat wave risk assessment of pregnant women

**DOI:** 10.1007/s00484-024-02738-x

**Published:** 2024-08-29

**Authors:** Ashish KC, Sujeena Maharjan, Omkar Basnet, Honey Malla, Rejina Gurung, Sunil Mani Pokharel, Gyanu Kumari Ghimire, Masoud Vaezghasemi, Julia Schröders

**Affiliations:** 1 School of Public Health and Community Medicine, University of Gothenburg, Medicinaregatan 18, Gothenburg 43190, Sweden; 2 Research Division, Golden Community, Jawgal, Lalitpur, Nepal; 3 Department of Women’s and Children’s Health, Uppsala University, Dag Hammarskjölds väg 14B, Uppsala, Sweden; 4 Bharatpur Hospital, Ministry of Health and Population, Chitwan, Nepal; 5 Department of Epidemiology and Global Health, Umeå University, Umeå 90187, Sweden

**Keywords:** Heatwaves, Pregnant women, Risk assessment, Construct validation, Reliability assessment, Nepal

## Abstract

Introduction: The 1.2 °C rise of global ambient temperature since the pre-industrial era has led to an increase the intensity and frequency of heatwaves. Given the heightened vulnerability of pregnant women to heat stress, there is an urgent need for tools which accurately assess the knowledge, risk, and perception of pregnant woman toward heatwaves, enabling effective policy actions. In this research, we developed and validated tools to evaluate pregnant women’s perceptions of heat wave risks and behaviors. Method: We developed 50 items across seven constructs using the Health Belief Model, identified through a systematic literature review. The constructs comprised 8 Knowledge(K) items, 4 in Perceived Vulnerability (PV), 5 in Perceived Severity (PS), 6 in Perceived Benefit (PB), 4 in Perceived Barrier (PBa), 5 in Cue to Action(Cu) and 18 in Adaptation(A). Cognitive testing was performed with a separate group of pregnant women(*n* = 20). The tested tools were then administered to 120 pregnant women residing during the spring-summer 2023. Construct validation utilized exploratory factor analysis. Results: The Principal Axis Factoring Method was employed in the EFA with oblimin rotation for 51 items, considering communality > 0.20, and aiming to extract three factors. Across the three factors with Cronbach’s alpha > 0.70, a total of 11 items were distributed. Factor 1 included Perceived Severity (PS1, PS2, PS3 and PS5); Factor 2 included Cue to Action (Cu1, Cu2, Cu3, and Cu4); and Factor 3 encompassed Perceived Vulnerability (PV1, PV2, PV4). Only two of the retained items had factor loadings > 0.50, namely PV4 and PS5. Consequently, the three constructs measuring Perceived Severity, Cues to Action, and Perceived Vulnerability using the HBM among pregnant women were deemed valid. Conclusion: Our study has successfully validated a highly reliable tool which stands ready for application in assessing pregnant women’s risk perception regarding heatwaves.

**Supplementary Information:**

The online version contains supplementary material available at 10.1007/s00484-024-02738-x.

## Introduction

In comparison to the baseline period of 1981–2010, projections indicate that the annual average temperature in the hotspots of South Asia is expected to increase by 1.5–3.0 °C by the year 2050 (Paola A. Arias 2021; Ma and Yuan 2021). There may be significant heat-related health consequences because more than half of the region’s population, or 800 million people reside in this area (Howard and Krishna 2022; Jain and Jain 2022; Luthi et al. 2023). Over the past 15 years, Nepal has witnessed a series of catastrophic weather events, with the Terai region emerging as the hottest part of the country (Kiarsi et al. 2023a). The prevalence of warm days and nights is on the rise in Nepal’s districts (Karki et al. 2016). According to the Department of Hydrology and Meteorology, June 2023 marked the hottest month ever recorded in Nepal’s history (National Oceanic and Atmospheric Administration 2021).

Several environmental factors and heatwaves have detrimental effects on maternal and fetal health, with the risk of adverse outcomes rising as ambient temperatures deviate from the optimal range (Kuehn and McCormick 2017; Ravanelli et al. 2019; Zinia et al. 2023). Several studies have indicated an increase of preterm births in geographical locations where pregnant woman are exposed to high ambient temperatures or heatwaves (He et al. 2016; Zheng et al. 2018; Yu et al. 2018; McElroy et al. 2022). A meta-analysis of 47 studies showed that per every 1.0-degree Celsius increase in temperature, the odds of preterm birth increased by 1.05 and 1.16-fold during heatwaves (Chersich et al. 2020). Heatwaves exert not only acute effects on maternal and fetal health but also influence fetal growth (Massimiliano Bratti May 2021). Numerous studies have established an association between higher temperatures and lower birth weight (Lawlor et al. 2005; Sun et al. 2019). Furthermore, for every 1-degree Celsius increase in temperature, the risk of a stillbirth rises by 1.05 times (Strand et al. 2012; Chersich et al. 2020). During periods of extreme heat, pregnant women may exhibit an increased frequency of visits to the emergency room and a higher incidence of cardiovascular events such as myocardial infarction and stroke (Davis and Novicoff 2018). During pregnancy, exposure to heat waves lead to physical and psychological stress, which results in increased systemic cortisol, subsequently activating two different physiological pathways (Hunter et al. 2023). First, the premature activation of cyclooxygenase-2 (COX-2) in the fetal membranes and placental membrane leads to premature labor and preterm birth. Second, increased systemic inflammation leads to the suppression of growth hormones and insulin-like growth factors (IGFs) resulting in growth restriction (Bonell et al. 2022; Hunter et al. 2023).

Pregnant women are notably excluded from the definition of “heat-susceptible individuals” as defined in the existing research. This literature primarily concentrates on vulnerable groups, including the poor, elderly, young children, minority groups, outdoor workers, individuals with chronic respiratory or cardiovascular conditions, socially isolated individuals and those residing in urban heat environments (Harlan et al. 2006). The broader public appears to have limited awareness of the consequences of heat exposure during pregnancy (Beckmann et al. 2021). People’s perceptions of the risk associated with natural disasters and their vulnerability to heat-related morbidity and mortality can be influenced by a number of factors such as age, health literacy and housing conditions (Naughton et al. 2002; Gil Cuesta et al. 2017). However, with increased awareness of these risks and the adoption of minor behavioral changes, individuals can significantly mitigate the likelihood of heat-induced illnesses and fatalities (Smoyer-Tomic and Rainham 2001). Data on perceived vulnerability, perceived severity and cues to action towards heat wave among pregnant women is not available.

This study aims to develop and validate a tool to assess heat wave knowledge, risk perceptions, vulnerability, and adaptive behavior among pregnant women. Its findings will inform the design of interventions aimed at enhancing adaptive behaviors among pregnant women, ultimately mitigating the impact of heatwaves on fetal growth and pregnancy outcomes, with lasting benefits for overall child and women’s health.

## Materials and methods

### Study design

A cross-sectional design utilizing a questionnaire-based survey was employed to collect information from pregnant women at Bharatpur hospital, Chitwan, Nepal, between August and October 2023. Situated in province 3, Bharatpur hospital is a provincial referral facility with 600 beds, recording 14,188 annual admission and11,820 annual delivery (Kc et al. 2019; Gurung et al. 2019). The medical staff includes three obstetricians, eleven medical officers, 22 midwives, eight skilled birth attendants (SBA), and 40 nursing and SBA students.

### Study setting

Chitwan, located in the Southern Flatland of Nepal near the equatorial belt (Karki et al. 2016), experienced temperatures ranging from 34.0 to 42.0 degrees Celsius between April and August 2023. In September and October 2023, temperatures remained between 31.0 and 38.0 degrees Celsius. In 2023, between April to October number of days and nights with temperature above 35.0 degree was 19 days and had increased by 16 days from that of 2000. The incremental increase in the number of hot days had increased by 0.65 days each year in Chitwan district.

Chitwan district is mostly characterized by flat terrain with some border regions featuring mountains. The area of green vegetation in this district is 1541 square kilometers and with rapid urbanization the green vegetation is in decline. The total population of the district according to the latest census 2021 is 719,895 with 325 population per square kilometer (Government of Nepal 2021). The rapid urbanization of the district has led to increase in migration from other districts. The 2022 annual estimated number of pregnant women for the Chitwan district is 11,662 with more than 95.0% seeking care from health institutions for pregnancy and childbirth (District Public Health Office 2022).

### Study population

The study employed a convenience sampling technique to recruit pregnant women from the gynecological ward at Bharatpur Hospital. Inclusion criteria encompassed pregnant women in their third trimester holding an ANC card, residing permanently in urban or rural areas with a travel time of not more than 90 min between home and hospital, and those who willingly provided consent to participate in the study. Out of 462 pregnant women admitted to the hospital in their third trimester, 264 met the eligibility criteria for participation, and 120 willingly consented to take part in the study.

### Data collection

An independent team of data collectors were trained to use the tool to interview the women who were enrolled in the study. The data collectors were trained to use a tablet-based application to record the interview responses. The data collectors also collected additional data on socio-demographic, housing and green space information together with heat risk perception.

### Data management

The data entered into the tablet-based application underwent a thorough review by an independent database manager. Subsequent to this verification, a detailed discussion was held with the data collector. To facilitate data cleaning, it was extracted into SPSS (IBM SPSS statistic software for Windows version 26.0). Prior to the analysis, data consistency was ensured, and any mismatches were identified and corrected accordingly.

### Development, validation, and reliability assessment of the data collection tool

We employed a systematic approach in the development of the data collection tool, encompassing validation and reliability assessments.

#### Step1: Literature review to identify relevant tools for assessing heatwave knowledge, risk perception, and adaptation

A comprehensive literature review was conducted to identify tools applied in the assessment of heatwave knowledge, risk perception, and adaptation. The systematic search involved querying original research studies focusing on these aspects. Our initial MEDLINE search utilized key terms “Heatwave” AND “Pregnant women” AND (“Risk Perception” OR “Risk knowledge” OR “Adaptation”). Four studies were identified using these search terms. Subsequently, we refined the search strategy by updating the terms to “Heatwave” AND “Vulnerable Population” AND (“Risk Perception” OR “Risk knowledge” OR “Adaptation”). This revised search yielded 13 studies employing tools and constructs to assess heatwave risk knowledge, perception, and adaptation (supplementary file 1).

#### Step 2: Development of constructs and items through expert review consultation

Based on 13, relevant studies (Liu et al. 2013; Akompab et al. 2013; Khare et al. 2015; Sayili et al. 2022), experts developed constructs and items to assess the knowledge, risk perception, and adaptation of pregnant women. The questionnaire comprised six parts, totaling 71 items (supplementary file 2). The obstetrical and health-related section featured three items, with information extracted from the ANC card. The socio-demographic and housing conditions sections included 16 items, and the green space section contained two items. The socio- demographic information included- maternal age in complete years; completed gestational age in weeks, medical complications during pregnancy categorized as none, gestational diabetes, low back pain and other; previous pregnancy; location of residence categorized as a rural and urban municipality (administrative region); caste categorized as advantaged (Brahmin, Chettri and Newar) and disadvantaged (Janjati, Dalit and Muslim) and primary education defined as 5 years or more.

For the knowledge, perception and adaptive behavior constructs (Heard et al. 2020), 50 items were created. The knowledge constructs consisted of three options per item with a total of three negative questions. Positive statements were scored as follows: 1 for True, 0 for False, and 0 for Don’t know. Negative statements were scored as: 1 for False, 0 for True, and 0 for Don’t Know. Using the Health Belief Model (HBM), the perception construct was divided into five parts: perceived vulnerability, perceived severity, perceived benefits, perceived barriers, and cues to action. Responses for perceived benefits, including three negative statements, were assessed on a 5-point Likert scale. For positive statements: 1 for strongly disagree, 2 for disagree, 3 for unknown, 4 for agree, and 5 for strongly agree. For negative statements: 1 for strongly agree, 2 for agree, 3 for unknown, 4 for disagree, and 5 for strongly disagree. The adaptive behavior construct included three options. Positive statements were scored as follows: 3 for always, 2 for sometimes, and 1 for never. Negative statements were scored as 1 for always, 2 for sometimes, and 3 for never. This construct comprised three negative statements. All information, excluding obstetrical and health-related data, was collected through face-to-face interviews utilizing a self-developed semi-structured questionnaire integrated into a tablet-based application.

#### Step 3: Content validity of constructs and items

Constructs and items were generated through a meticulous process, involving an in-depth literature search and consistent collaboration with experts. To ensure content validity, a series of three meetings were conducted with a multidisciplinary team compromising epidemiologists, obstetricians, nurses, midwives, pediatricians, and data scientists. These sessions were dedicated to the thorough review and validation for the developed tools.

#### Step 4- Cognitive testing of the constructs and items

The developed constructs and items, along with demographic, health and green space information, were translated to Nepali. The English version of the tool underwent translation to Nepali, and the Nepali version was subsequently back-translated into English by an independent translator. To assess cognitive understanding, testing was conducted among 20 pregnant women at the hospital from July 17–19, 2023. Despite repeated attempts, the cognitive examination revealed difficulties among responders in comprehending questions ‘201’ within the knowledge construct and ‘705’ within the cues to action construct (supplementary file 3).

#### Step 5- Construct validity assessment of constructs and items using exploratory factor analysis

The construct validity of the Knowledge, Perception and Adaptive behavior constructs, comprising of a total of 50 items, was evaluated through exploratory factor analysis based on data generated from the pilot study involving 120 pregnant women. Since the scale lacked prior validation, and the relationship between variables and factors were unknown, exploratory factor analysis was used for variable assignment into factors. The assessment involved the following steps: (i) analysis of the bivariate correlation matrix of the 50 items, where high correlation values (> 0.80) resulted in the deletion of one item from pairs exhibiting multicollinearity; (ii) application of the Kaiser–Meyer–Olkin test (KMO) to test the sample adequacy (KMO value above 0.50 indicating adequate sampling for factor analysis) and Bartlet’s test of Specificity (Bartlet’s test below 0.05 indicating suitability for factor analysis); (iii) execution of exploratory factor analysis for all seven constructs using the Principal Axis Factoring Method with Varimax rotation; (iv) checking communalities of the items, removing those below 0.20, and rerunning the analysis; (v) analyzing the rotated factor matrix and suppressing items with factor loadings below 0.40; and removing factors without at least three items with acceptable factor loading (≥ 4.0) and rerunning the analysis; (vi) eliminating items with cross-loadings > 75% and rerunning the analysis; (vii) stabilizing the final solution of the rotated factor matrix by meeting all previously set requirements; (viii) running principal component analysis (PCA) for each identified factor to compute the overall average correlation within the factor, and regression scores were recorded. Utilizing these regression scores, the average correlation between the factors were calculated; (ix) computing the differences between the overall within and between factor correlation. Since the difference was not significantly higher, Principal Axis Factoring with Oblique rotation with delta equal to 0 was carried out for all 50 items.

The KMO statistics of the final solution (Pattern Matrix) were verified (> 0.50), and the determinant of the correlation matrix should be > 0.01. The total variance explained by the retained factors should be at least 50.0%, and the communalities of the final solution (Pattern Matrix) for sample size 100–200 fall between 0.50 and 0.60. PCA was conducted for each of the retained three factors separately, ensuring that the Eigen value of each component was greater than zero, indicating a robust solution.

#### Step 6- Reliability of constructs and items

The internal consistency of the Knowledge, Perception and Adaptive behavior constructs was assessed by calculating Cronbach’s alpha. A Cronbach’s alpha value above 0.80 is indicative of good internal consistency. Following the reliability analysis, the interpreted items were examined, and names were assigned to the constructs.

### Ethics

The study received approval from the Institutional Review Board (IRC) of Bharatpur Hospital, Chitwan, Nepal. Every participant was provided with comprehensive information about the study, and written consent was obtained before the commencement of data collection.

## Result

The results are based on the complete data from 120 women/mothers/pregnant women. The mean and standard deviation of maternal age was 26.5 ± 4.8 and mean gestational week (SD) was 37.5 ± 3.3 week. Among the participants, 81.7% of them didn’t have any medical complications; 87.5% of the participants were pregnant for the first time; 73.3% of the pregnant women live in urban area (urban municipality); 68.3% of the pregnant women were from disadvantageous caste group; 96.7% of the participants attended the school and 51.7% had tin roof (Table 1).

**Table 1:** Characteristics of the study population (*n* = 120)

Variable	
	Mean ± S.D
Maternal age	26.5 ± 4.8
Completed gestational week	37.5 ± 3.3
Medical Complications during pregnancy*	**N (%)**
None	98 (81.7)
Gestational Diabetes	4 (3.3)
Low back pain	6 (5)
Others	12 (10)
**Parity**,** previous pregnancy**	**N (%)**
Primi para	105 (87.5)
Multi-para	15 (12.5)
**Location of residence**	**N (%)**
Rural	32 (26.7)
Urban	88 (73.3)
**Caste**	**N (%)**
Advantaged**	38 (31.7)
Disadvantaged***	82 (68.3)
**Education**	**N (%)**
Yes	116 (96.7)
No	4 (3.3)

*multiresponse; **Advantaged caste- Brahmin, Chettri and Newar; *** disadvantaged caste- Janjati, Dalit, Muslim

### Validity test of knowledge, perceptions and adaptive behavior by exploratory factor analysis

**Fig. 1:**
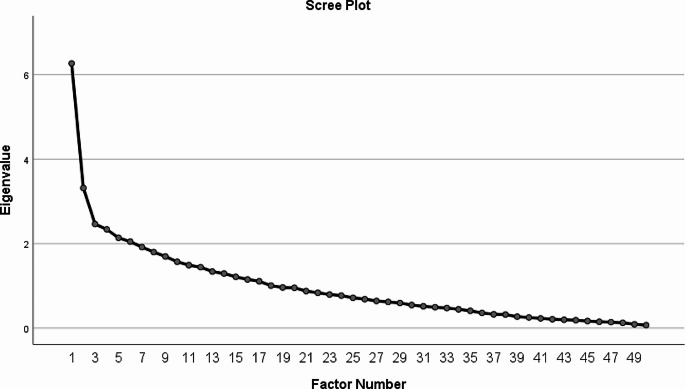
Scree plot

The bivariate correlation matrix of all the items was analyzed. None of the pair of items had the correlation greater than 0.80 (Supplementary Table 1). The KMO index for the construct Knowledge, Perceptions and Adaptive behavior was 0.58 indicating the sample adequacy for factor analysis. The Bartlett test of specificity was significant (supplementary Table 2). At the beginning of the study, there were 7 constructs. Exploratory factor analysis was conducted using the Principal Axis Factoring Method with Varimax rotation. To have an optimal solution 100 iterations were done. The scree plot of the exploratory analysis showed 3 factors to be extracted. (Fig. 1)

In the initial solution none of the items had communalities less than 0.20. The items having communalities less than 0.20 were removed and rerun in further solution (Supplementary Table 3). Only factors with at least 3 items with acceptable factor loading (correlation of each item with the factor) of with low cross loading were retained. The items with a factor loading < 0.40 were removed and rerun until it gave the stable solution by fulfilling all the criteria. Finally, after 41 steps, the EFA gave the stable solution of 3 factors fulfilling all requirements.

Principal Axis Factoring Method with varimax rotation gave the following factors and items. In factor 1, items PV3, PS1, PS2, PS3 and PS5; in factor 2, items Cu1, Cu2, Cu3 and Cu4 and in factor 3, items PV1, PV2 and PV4.

Factor 1 had 5 Items; Factor 2 had four items and Factor 3 with 3 items. The final stabilized solution consists of 3 factors with 12 items. There were cross loading of an item PV3 in factor 1 and 3, an item PV4 in factor 1 and 3. But both the item PV3 and PV4 has cross loading < 75.0%, so it was included in the factor 1 and 3, respectively (Table 2).

**Table 2:** Rotated factor Matrix

Item	Factor		
	1	2	3
**PV1**			0.82
**PV2**			0.76
**PV3**	0.52		0.41
**PV4**	0.58		0.67
**PS1**	0.70		
**PS2**	0.67		
**PS3**	0.86		
**PS5**	0.43		
**Cu1**		0.71	
**Cu2**		0.76	
**Cu3**		0.84	
**Cu4**		0.82	

The 1st, 2nd and 3rd factor represents 20.2%, 18.3%, and 16.0% of total variance, respectively (Table 3).

**Table 3:** Total variance explained by three factors

Initial Eigenvalues				Extraction Sums of Squared Loadings			Rotation Sums of Squared Loadings		
Factor	Total	% variance	Cumulative %	Total	% of variance	Cumulative %	Total	% of Variance	Cumulative %
1	5.2	36.9	36.9	4.82	34.4	34.4	2.8	20.2	20.2
2	2.6	18.4	55.4	2.22	15.9	50.3	2.6	18.3	38.4
3	1.0	7.4	62.7	0.58	4.1	54.5	2.3	16.0	54.5
4.	1.0	7.1	69.8						
5.	0.86	6.1	75.9						
6.	0.75	5.3	81.2						
7.	0.52	3.7	84.9						
8.	0.49	3.5	88.4						
9.	0.42	3.0	91.4						
10	0.33	2.4	93.8						
11	0.30	2.2	95.9						
12	0.26	1.8	97.8						
13.	0.18	1.3	99.0						
14.	0.14	0.98	100.0						

When PCA was run for each factor and the average correlation within each factor and the overall average correlation within the factors were calculated (Supplementary file 4). The average correlation within each factor; for factor 1 it was 0.66; for factor 2 it was 0.78 and for factor 3 it was 0.87. The overall average within factor correlation was 0.79 (Supplementary Table 4). The average correlation between the factors were calculated in by downloading the regression scores (Table 4). The average correlation within factors was slightly higher than the average correlation between factors, and thus not acceptable.

**Table 4:** Correlation between factors

	Factor 1	Factor 2	Factor 3
Factor 1	1.0		
Factor 2	0.23	1.0	
Factor 3	0.72	0.22	1.0
Average	0.70		

Again, the Principal Axis Factoring with the direct Oblimin oblique rotation was done with delta equal to 0 for 50 items. In the initial solution, none of the items had communalities less than 0.20. After 37 steps, it yielded a stable solution with 3 factors and 11 items. Factor 1 had PS1 (0.75), PS2 (0.69), PS3 (0.94) and PS5 (0.43) items; factor 2 had Cu1 (0.71), Cu2 (0.76), Cu3 (0.84) and Cu4 (0.84) items; and the factor 3 had PV1 (0.71), PV2 (0.65) and PV4 **(**0.43) items. Item PV4 had cross-loading in the factor 1 and 3. The requirements set above stated that there must be at least 3 items in a factor and the cross loading > 75.0% is not acceptable. Item PV4 had cross loading of > 75.0% in factor 1 and 3, and thus acceptable. It was included in the Factor 3 to fulfill the requirements of at least 3 items per factor (Table 5).

**Table 5:** Pattern matrix (final solution)

Items	Factor 1	Factor 2	Factor 3
**PV1**			0.71
**PV2**			0.65
**PV4**	0.61		0.43
**PS1**	0.75		
**PS2**	0.69		
**PS3**	0.94		
**PS5**	0.43		
**Cu1**		0.71	
**Cu2**		0.76	
**Cu3**		0.84	
**Cu4**		**0.**84	

The KMO value for the final solution was 0.80 (Supplementary Table 5). In terms of reliability testing, the Cronbach’s alpha for factor 1, factor 2 and factor 3 were 0.79, 0.87 and 0.90 respectively (Table 6). The three scales had acceptable Cronbach’s alpha, Eigen value and acceptable factor loading. Finally, the scales were interpreted qualitatively and given appropriate names. Factor 1, Factor 2, and Factor 3 were as named Perceived Severity, Cues to action and Perceived Vulnerability, respectively.

**Table 6:** Reliability study of the retained items

Factors	Items	Cronbach alpha	Overall Cronbach alpha
1	PS1, PS2, PS3, PS5	0.79	0.85
2	Cu1, Cu2, Cu3, Cu4	0.87	
3	PS1, PS2, PS3	0.90	

## Discussion

Despite pregnant women meriting distinct considerations in heatwave risk assessment due to their unique physiological vulnerabilities to heat stress (Hunter et al. 2023) and the association of heat exposure with adverse acute and long-term fetal (Bonell et al. 2022) and child health outcomes (Massimiliano Bratti May 2021), they have limited representation in the existing literature (Samuels et al. 2022). In response to this gap, the current study has developed a comprehensive scale and items with validated and reliable measures for assessing heatwave-related factors, including perceived severity, perceived vulnerability, and cues to action specifically among pregnant women. Understanding women’s knowledge, risk perception, and adaptive behaviors during heatwaves is crucial for informed decision-making and effective healthcare interventions. Designing tools like ours that are tailored to assess heatwave risks in pregnant women not only provides a nuanced understanding of their experiences but also facilitate the implementation of targeted interventions to safeguard maternal and fetal health.

Based on our comprehensive literature search date, it became evident that there was a need to develop a scale with valid and reliable items for a heatwave tool. Our tool aims to assess Perceived Severity, Perceived Vulnerability, and Cues to Action among pregnant women. Despite several studies highlighting the adverse effects of heatwaves on pregnant women (Lin et al. 2017; Isen et al. 2017; Ravanelli et al. 2019), there is a notable absence of validated tools specifically designed to assess vulnerability, severity, and cues to action in this population. While some studies conducted in Turkey (Sayili et al. 2022), Malaysia (Arsad et al. 2022), and Iran (Kiarsi et al. 2023b) focused on psychometric properties related to the general population risk perception, only a study in Malaysia developed a tool for assessing risk perception among the general population. The evidence from these studies underscores the urgency of prioritizing protection for pregnant women from the impacts of heatwaves.

A study conducted in Australia indicated that individuals with high cues to action were more inclined to exhibit positive adaptation behaviors in response to heatwaves (Akompab et al. 2013). Events, stimuli, and people may have ​influenced their behavior regarding heatwaves, potentially driven by an awareness of the vulnerability and severity of health impacts. These findings emphasize the importance of assessing risk perception and cues to action specifically among pregnant women.

The findings regarding Cues to Action underscore the pivotal role of social networks in shaping pregnant women’s responses to heatwaves. The study revealed that family, friends, media, and healthcare providers emerged as influential sources of information, acting as cues to action for protective measures against heatwaves. The presence of supportive networks and the dissemination of information through various channels not only enhance awareness but also contribute significantly to the adoption of adaptive behaviors. These results emphasize the need to harness and leverage existing social structures and communication channels in crafting effective public health strategies to mitigate the impact of heatwaves on pregnant women. Public health campaigns often utilize various communication channels, including social networks, to raise awareness, provide guidance, and encourage protective measures (Hunter et al. 2019). Tailoring interventions to leverage social networks, such as involving family, friends, and healthcare providers, can enhance the effectiveness of efforts to mitigate the impact of heatwaves, especially among vulnerable populations like pregnant women. To date, there has been a scarcity of social interventions addressing heatwaves, particularly within heat-prone low- and middle-income country (LMIC) contexts, and virtually none targeting pregnant women (Mayrhuber et al. 2018; Roos et al. 2021; Conway et al. 2024). Our study has the potential to catalyze the creation of social network-based interventions aimed at enhancing heatwave resilience among vulnerable pregnant women.

Hence, our tool can be employed to assess perceived vulnerability, perceived severity, and cues to action among pregnant women. The results of this study will play a crucial role in shaping the formulation and execution of heatwave adaptation policies tailored for pregnant women. Consequently, these policies are anticipated to mitigate vulnerability and severity to the effects of heatwaves by enhancing adaptive behaviors among the pregnant women.

Limitations.

There are several limitations to the study. First, there is a selection bias in the recruitment of participants. The participants included in the study may not be representative of the general population as we included only those women who came to the public hospital for pregnancy care. However, the distribution of disadvantaged to advantaged populations (68.3–31.7%) among the women coming to the hospital was similar to the national population distribution (67.6–32.4%) (National Statistic Office 2021). The second limitation is the interviewer bias which might have been introduced as there were two trained interviewers interviewing all pregnant women. To reduce the interviewer bias, the project coordinator (OB) conducted regular training for the interviewers. The third limitation is recall bias from the pregnant women, especially in terms of household information or information related to adaptation to heatwave.

## Conclusion

This study systematically developed a tool to assess pregnant women’s Perceived vulnerability, Perceived severity, and Cues to action during heatwaves using the Health Belief Model. The tool’s construct, content validity and reliability were tested using novel statistical methods, making it suitable for assessing heat wave risk perception among pregnant women in various settings. In Nepal, the tool can be used to assess pregnant women’s heat wave risk perception without additional cognitive testing, while in other settings cognitive testing is required. Furthermore, this research sheds lights on the factors, events, and triggers influencing pregnant women’s behavior in response to heatwaves, providing valuable information for planners and policymakers to enhance and refine adaptation policies. Particularly, Insights into the data on vulnerability and severity experienced by pregnant women offer a foundation for formulating effective adaptation policies.

## Electronic supplementary material

Below is the link to the electronic supplementary material.

Supplementary file 1

Supplementary file 2

Supplementary file 3

Supplementary table 1- 5

Supplementary file 4

## Abbreviations

KMO: Kaiser-Meyer-Olkin
PS: Perceived susceptibility
Cu: Cue for Action
PV: Perceived vulnerability
PB: Perceived barrier

## References

[CR1] Akompab DA, Bi P, Williams S, Grant J, Walker IA, Augoustinos M (2013) Heat waves and climate change: applying the health belief model to identify predictors of risk perception and adaptive behaviours in adelaide, Australia. Int J Environ Res Public Health 10(6):2164–2184. 10.3390/ijerph10062164 23759952 10.3390/ijerph10062164 PMC3717730

[CR2] Arsad FS, Hod R, Ahmad N, Baharom M, Tangang F (2022) The malay-version knowledge, risk perception, attitude and practice questionnaire on heatwaves: Development and Construct Validation. Int J Environ Res Public Health 19(4). 10.3390/ijerph19042279 10.3390/ijerph19042279 PMC8872578 35206467

[CR3] Beckmann SK, Hiete M, Schneider M, Beck C (2021) Heat adaptation measures in private households: an application and adaptation of the protective action decision model. Hum Soc Sci Commun 8(1). 10.1057/s41599-021-00907-6

[CR4] Bonell A, Sonko B, Badjie J, Samateh T, Saidy T, Sosseh F, Sallah Y, Bajo K, Murray KA, Hirst J, Vicedo-Cabrera A, Prentice AM, Maxwell NS, Haines A (2022) Environmental heat stress on maternal physiology and fetal blood flow in pregnant subsistence farmers in the Gambia, West Africa: an observational cohort study. Lancet Planet Health 6(12):e968–e976. 10.1016/S2542-5196(22)00242-X 36495891 10.1016/S2542-5196(22)00242-X PMC9756110

[CR5] Chersich MF, Pham MD, Areal A, Haghighi MM, Manyuchi A, Swift CP, Wernecke B, Robinson M, Hetem R, Boeckmann M, Hajat S, Grp CCH-HS (2020) Associations between high temperatures in pregnancy and risk of preterm birth, low birth weight, and stillbirths: systematic review and meta-analysis. Bmj-Brit Med J 371. 10.1136/bmj.m3811 10.1136/bmj.m3811 PMC7610201 33148618

[CR6] Conway F, Portela A, Filippi V, Chou D, Kovats S (2024) Climate change, air pollution and maternal and newborn health: an overview of reviews of health outcomes. J Glob Health 14:04128. 10.7189/jogh.14.04128 38785109 10.7189/jogh.14.04128 PMC11117177

[CR7] Davis RE, Novicoff WM (2018) The Impact of Heat Waves on Emergency Department Admissions in Charlottesville, Virginia, U.S.A. Int J Environ Res Public Health 15 (7). 10.3390/ijerph15071436 10.3390/ijerph15071436 PMC6068980 29986505

[CR35] District Public Health Office (2022) Annual Health Report. Chitwan, Nepal

[CR8] Gil Cuesta J, van Loenhout JA, Colaco MD, Guha-Sapir D (2017) General Population Knowledge about Extreme Heat: a cross-sectional survey in Lisbon and Madrid. Int J Environ Res Public Health 14(2). 10.3390/ijerph14020122 10.3390/ijerph14020122 PMC5334676 28134849

[CR9] Government of Nepal (2021) National Statistic Office. Kathmandu, Nepal

[CR10] Gurung R, Jha AK, Pyakurel S, Gurung A, Litorp H, Wrammert J, Jha BK, Paudel P, Rahman SM, Malla H, Sharma S, Gautam M, Linde JE, Moinuddin M, Ewald U, Målqvist M, Axelin A, Ashish KC (2019) Scaling up Safer Birth Bundle through Quality Improvement in Nepal (SUSTAIN)a stepped wedge cluster randomized controlled trial in public hospitals. Implement Sci 14. 10.1186/s13012-019-0917-z 10.1186/s13012-019-0917-z PMC6582583 31217028

[CR11] Harlan SL, Brazel AJ, Prashad L, Stefanov WL, Larsen L (2006) Neighborhood microclimates and vulnerability to heat stress. Soc Sci Med 63(11):2847–2863. 10.1016/j.socscimed.2006.07.030 16996668 10.1016/j.socscimed.2006.07.030

[CR12] He JR, Liu Y, Xia XY, Ma WJ, Lin HL, Kan HD, Lu JH, Feng Q, Mo WJ, Wang P, Xia HM, Qiu X, Muglia LJ (2016) Ambient temperature and the risk of Preterm Birth in Guangzhou, China (2001–2011). Environ Health Perspect 124(7):1100–1106. 10.1289/ehp.1509778 26672059 10.1289/ehp.1509778 PMC4937853

[CR13] Heard E, Fitzgerald L, Wigginton B, Mutch A (2020) Applying intersectionality theory in health promotion research and practice. Health Promot Int 35(4):866–876. 10.1093/heapro/daz080 31390472 10.1093/heapro/daz080

[CR14] Howard S, Krishna G (2022) How hot weather kills: the rising public health dangers of extreme heat. Bmj-Brit Med J 378. 10.1136/bmj.o1741 10.1136/bmj.o1741 35835453

[CR16] Hunter RF, de la Haye K, Murray JM, Badham J, Valente TW, Clarke M, Kee F (2019) Social network interventions for health behaviours and outcomes: a systematic review and meta-analysis. PLoS Med 16(9):e1002890. 10.1371/journal.pmed.1002890 31479454 10.1371/journal.pmed.1002890 PMC6719831

[CR15] Hunter PJ, Awoyemi T, Ayede AI, Chico RM, David AL, Dewey KG, Duggan CP, Gravett M, Prendergast AJ, Ramakrishnan U, Ashorn P, Klein N, St LSVN (2023) Biological and pathological mechanisms leading to the birth of a small vulnerable newborn. Lancet 401(10389). 10.1016/S0140-6736(23)00573-1 10.1016/S0140-6736(23)00573-1 37167990

[CR18] Isen A, Rossin-Slater M, Walker R (2017) Relationship between season of birth, temperature exposure, and later life wellbeing. Proc Natl Acad Sci U S A 114(51):13447–13452. 10.1073/pnas.1702436114 29203654 10.1073/pnas.1702436114 PMC5754756

[CR19] Jain Y, Jain R (2022) India and Pakistan emerge as early victims of extreme heat conditions due to climate injustice. Bmj-Brit Med J 377. 10.1136/bmj.o1207 10.1136/bmj.o1207 35562114

[CR20] Karki R, Talchabhadel R, Aalto J, Baidya SK (2016) New climatic classification of Nepal. Theor Appl Climatol 125(3–4):799–808. 10.1007/s00704-015-1549-0

[CR21] Kc A, Bergström A, Chaulagain D (2019) Scaling up quality improvement intervention for perinatal care in Nepal (NePeriQIP); study protocol of a cluster randomised trial (2, e000497, 2017). Bmj Global Health 4(6). 10.1136/bmjgh-2017-000497corr1 10.1136/bmjgh-2017-000497 PMC5640082 29071130

[CR22] Khare S, Hajat S, Kovats S, Lefevre CE, de Bruin WB, Dessai S, Bone A (2015) Heat protection behaviour in the UK: results of an online survey after the 2013 heatwave. BMC Public Health 15:878. 10.1186/s12889-015-2181-8 26357923 10.1186/s12889-015-2181-8 PMC4566312

[CR23] Kiarsi M, Amiresmaili M, Mahmoodi MR, Farahmandnia H, Nakhaee N, Zareiyan A, Aghababaeian H (2023a) Heat waves and adaptation: a global systematic review. J Therm Biol 116:103588. 10.1016/j.jtherbio.2023.103588 37499408 10.1016/j.jtherbio.2023.103588

[CR24] Kiarsi M, Doustmohammadi MM, Reza Mahmoodi M, Nakhaee N, Zareiyan A, Aghababaeian H, Amiresmaili M (2023b) Development and validation of heat wave hazard adaptation tool: a study protocol. Disaster Emerg Med J 8(2):110–121. 10.5603/DEMJ.a2022.0038

[CR25] Kuehn L, McCormick S (2017) Heat exposure and Maternal Health in the Face of Climate Change. Int J Environ Res Public Health 14(8). 10.3390/ijerph14080853 10.3390/ijerph14080853 PMC5580557 28758917

[CR26] Lawlor DA, Leon DA, Davey Smith G (2005) The association of ambient outdoor temperature throughout pregnancy and offspring birthweight: findings from the Aberdeen Children of the 1950s cohort. BJOG 112(5):647–657. 10.1111/j.1471-0528.2004.00488.x 15842292 10.1111/j.1471-0528.2004.00488.x

[CR27] Lin Y, Hu W, Xu J, Luo Z, Ye X, Yan C, Liu Z, Tong S (2017) Association between temperature and maternal stress during pregnancy. Environ Res 158:421–430. 10.1016/j.envres.2017.06.034 28689033 10.1016/j.envres.2017.06.034

[CR28] Liu T, Xu YJ, Zhang YH, Yan QH, Song XL, Xie HY, Luo Y, Rutherford S, Chu C, Lin HL, Ma WJ (2013) Associations between risk perception, spontaneous adaptation behavior to heat waves and heatstroke in Guangdong province, China. BMC Public Health 13:913. 10.1186/1471-2458-13-913 24088302 10.1186/1471-2458-13-913 PMC3853971

[CR29] Luthi S, Fairless C, Fischer EM, Scovronick N, Ben A, Coelho M, Guo YL, Guo Y, Honda Y, Huber V, Kysely J, Lavigne E, Roye D, Ryti N, Silva S, Urban A, Gasparrini A, Bresch DN, Vicedo-Cabrera AM (2023) Rapid increase in the risk of heat-related mortality. Nat Commun 14(1):4894. 10.1038/s41467-023-40599-x 37620329 10.1038/s41467-023-40599-x PMC10449849

[CR30] Ma F, Yuan X (2021) Impact of climate and population changes on the increasing exposure to summertime compound hot extremes. Sci Total Environ 772:145004. 10.1016/j.scitotenv.2021.145004 33770855 10.1016/j.scitotenv.2021.145004

[CR31] Massimiliano Bratti PBF, Simone Russo (2021) May Prenatal Exposure to Heat Waves and Child Health in Sub-saharan Africa. Discussion Paper Series. Bonn, Germany

[CR32] Mayrhuber EA, Duckers MLA, Wallner P, Arnberger A, Allex B, Wiesbock L, Wanka A, Kolland F, Eder R, Hutter HP, Kutalek R (2018) Vulnerability to heatwaves and implications for public health interventions - a scoping review. Environ Res 166:42–54. 10.1016/j.envres.2018.05.021 29859940 10.1016/j.envres.2018.05.021

[CR33] McElroy S, Ilango S, Dimitrova A, Gershunov A, Benmarhnia T (2022) Extreme heat, preterm birth, and stillbirth: a global analysis across 14 lower-middle income countries. Environ Int 158:106902. 10.1016/j.envint.2021.106902 34627013 10.1016/j.envint.2021.106902

[CR17] National Ocean and Atompheric Adminstration (NOAA), National Centers for Environmental Investigations (2021) Global surface Summary of the day– GSOD. NOAA, National Centers for Environmental Information. United States

[CR36] National Statistic Office (2021) National Report on Caste/Ethnicity, Language and Religion. National Population and Household Census 2021. Government of Nepal, Kathmandu

[CR34] Naughton MP, Henderson A, Mirabelli MC, Kaiser R, Wilhelm JL, Kieszak SM, Rubin CH, McGeehin MA (2002) Heat-related mortality during a 1999 heat wave in Chicago. Am J Prev Med 22(4):221–227. 10.1016/s0749-3797(02)00421-x 11988377 10.1016/s0749-3797(02)00421-x

[CR37] Paola A, Arias NB, Coppola E, Jones RG, Krinner G, Marotzke J, Naik V, Palmer MD, Plattner G-K, Rogelj J, Rojas M, Sillmann J, Storelvmo T, Thorne PW, Blair Trewin (2021) Technical Summary. In Climate Change 2021: The Physical Science Basis. Contribution of Working Group I to the Sixth Assessment Report of the Intergovernmental Panel on Climate Change. Cambridge University Press, United Kingdom and United States

[CR38] Ravanelli N, Casasola W, English T, Edwards KM, Jay O (2019) Heat stress and fetal risk. Environmental limits for exercise and passive heat stress during pregnancy: a systematic review with best evidence synthesis. Br J Sports Med 53(13):799–805. 10.1136/bjsports-2017-097914 29496695 10.1136/bjsports-2017-097914

[CR39] Roos N, Kovats S, Hajat S, Filippi V, Chersich M, Luchters S, Scorgie F, Nakstad B, Stephansson O, Consortium C (2021) Maternal and newborn health risks of climate change: a call for awareness and global action. Acta Obstet Gynecol Scand 100(4):566–570. 10.1111/aogs.14124 33570773 10.1111/aogs.14124

[CR40] Samuels L, Nakstad B, Roos N, Bonell A, Chersich M, Havenith G, Luchters S, Day LT, Hirst JE, Singh T, Elliott-Sale K, Hetem R, Part C, Sawry S, Le Roux J, Kovats S (2022) Physiological mechanisms of the impact of heat during pregnancy and the clinical implications: review of the evidence from an expert group meeting. Int J Biometeorol 66(8):1505–1513. 10.1007/s00484-022-02301-6 35554684 10.1007/s00484-022-02301-6 PMC9300488

[CR41] Sayili U, Siddikoglu E, Pirdal BZ, Uygur A, Toplu FS, Can G (2022) The heat wave knowledge, awareness, practice and behavior scale: scale development, validation and reliability. PLoS ONE 17(12):e0279259. 10.1371/journal.pone.0279259 36542649 10.1371/journal.pone.0279259 PMC9770401

[CR42] Smoyer-Tomic KE, Rainham DG (2001) Beating the heat: development and evaluation of a Canadian hot weather health-response plan. Environ Health Perspect 109(12):1241–1248. 10.1289/ehp.011091241 11748031 10.1289/ehp.011091241 PMC1240506

[CR43] Strand LB, Barnett AG, Tong S (2012) Maternal exposure to ambient temperature and the risks of preterm birth and stillbirth in Brisbane, Australia. Am J Epidemiol 175(2):99–107. 10.1093/aje/kwr404 22167749 10.1093/aje/kwr404

[CR44] Sun S, Spangler KR, Weinberger KR, Yanosky JD, Braun JM, Wellenius GA (2019) Ambient temperature and markers of fetal growth: a retrospective observational study of 29 million U.S. Singleton births. Environ Health Perspect 127(6):67005. 10.1289/EHP4648 31162981 10.1289/EHP4648 PMC6792370

[CR45] Yu X, Feric Z, Cordero JF, Meeker JD, Alshawabkeh A (2018) Potential influence of temperature and precipitation on preterm birth rate in Puerto Rico. Sci Rep-Uk 8. 10.1038/s41598-018-34179-z 10.1038/s41598-018-34179-z PMC6208375 30382121

[CR46] Zheng X, Zhang W, Lu C, Norback D, Deng Q (2018) An epidemiological assessment of the effect of ambient temperature on the incidence of preterm births: identifying windows of susceptibility during pregnancy. J Therm Biol 74:201–207. 10.1016/j.jtherbio.2018.04.001 29801628 10.1016/j.jtherbio.2018.04.001

[CR47] Zinia SS, Yang KH, Lee EJ, Lim MN, Kim J, Kim WJ, Ko CS (2023) Effects of heavy metal exposure during pregnancy on birth outcomes. Sci Rep 13(1):18990. 10.1038/s41598-023-46271-0 37923810 10.1038/s41598-023-46271-0 PMC10624662

