# Supplementary material for: Development, validation and reliability of scales and items for heat wave risk assessment of pregnant women
Source: Int J Biometeorol. 2024 Aug 29;68(11):2205–14. doi: 10.1007/s00484-024-02738-x (PMC11519304; doi:10.1007/s00484-024-02738-x)
Supplement: Supplementary file 2 [file 484_2024_2738_MOESM2_ESM.docx]

**Supplementary file 2. 50 items.** Perceptions, risks and adaptation of heat waves among pregnant women

*Part A. Knowledge regarding heat wave on pregnant woman*

|  | **Statement** | **True** | **False** | **I don’t know** |
| --- | --- | --- | --- | --- |
| K1 | Heat wave are less common in urban areas as compared to rural areas because of house's shade. | **0** | **0** | 1 |
| K2 | Pregnant women are particularly at risk during heat wave. | 1 | 0 | 0 |
| K3 | Excessive sweating during heat wave might be sign of heat stress. | 1 | 0 | 0 |
| K4 | Pregnant women with health problems are at risk of becoming sick during heat wave. | 1 | 0 | 0 |
| K5 | There is no evidence of heat waves causing premature birth. | **0** | **0** | 1 |
| K6 | Heat wave does not change food behavior during pregnancy. | **0** | **0** | 1 |
| K7 | Heat wave fosters development of harmful bacteria in water and food. | 1 | 0 | 0 |
| K8 | Heat stress is worse during night time than during day time. | **0** | **0** | 1 |

Part B- Perception of pregnant woman on heat wave using structure of Health Belief Model

|  | **Statements on perceptions (Five constructs of the HBM)** | | **Response** | | | | |
| --- | --- | --- | --- | --- | --- | --- | --- |
|  |  |  | **SA** | **A** | **U** | **D** | **SD** |
| PV1 | Perceived risk | I think I might get dehydrated during a heat wave. |  |  |  |  |  |
| PV2 |  | I think my body temperature might increase abnormally during a heat wave. |  |  |  |  |  |
| PV3 |  | I think I might suffer from body weakness during a heat wave |  |  |  |  |  |
| PV4 |  | I think I might get sun burn during a heat wave. |  |  |  |  |  |
| PS1 | Perceived severity | If my body temperature rises during heat a wave, I might have to consult a health care provider. |  |  |  |  |  |
| PS2 |  | If I get dehydrated during a heat wave, I might have to be admitted in hospital. |  |  |  |  |  |
| PS3 |  | Dehydration in such heat wave might cause long term damage to my health. |  |  |  |  |  |
| PS4 |  | If I get sun burn during a heat wave, it might harm my fetal growth. |  |  |  |  |  |
| PS5 |  | Hospitalization as a result of dehydration during heat wave might risk my pregnancy. |  |  |  |  |  |
| PB1 | Perceived benefit | Eating hot foods during hot wave will enable me to withstand the heat. |  |  |  |  |  |
| PB2 |  | Living in cool environment will reduce my risk of getting dehydrated. |  |  |  |  |  |
| PB3 |  | Using sun blocking curtain will protect me from getting sun burn during heat wave. |  |  |  |  |  |
| PB4 |  | Wearing dark clothes during heat wave will reduce my sweating. |  |  |  |  |  |
| PB5 |  | Listening to weather update daily will enable me to plan my outdoor works. |  |  |  |  |  |
| PB6 |  | Staying indoors during heatwave will be quite unpleasant. |  |  |  |  |  |
| PoB1 | Perceived barrier | Taking cold shower from time to time at home during heat wave will waste water and increase my water charge. |  |  |  |  |  |
| PoB2 |  | Due to security reason, I will not open doors at night time to allow air in during hot wave. |  |  |  |  |  |
| PoB3 |  | Due to my health status, I will drink less water during heat wave. |  |  |  |  |  |
| PoB4 |  | I will not turn on fan during heat wave as it increases my electricity bill. |  |  |  |  |  |
| Cu1 | Cues to action | Family members and friends tell me about the dangers of heat wave. |  |  |  |  |  |
| Cu2 |  | I watch television to see how ambulance takes person dehydrated due to heat wave to hospital. |  |  |  |  |  |
| Cu3 |  | I read local newspaper and get news about health effects of heat wave. |  |  |  |  |  |
| Cu4 |  | Health care provider aware me about dangers of heat wave during antenatal check-up. |  |  |  |  |  |
| Cu5 |  | Due to my personal experience in heat wave, I will be safe during heat wave. |  |  |  |  |  |

Part C- Adaptation by pregnant woman during heat wave

|  | **statement** | **Score** | | |
| --- | --- | --- | --- | --- |
|  |  | **Always** | **Sometimes** | **Never** |
| A1 | Drinks plenty of water to stay hydrated. |  |  |  |
| A2 | Wears dark clothes when going out. |  |  |  |
| A3 | Drinks few cups of hot drinks to stay alert. |  |  |  |
| A4 | Wears cap when going outside. |  |  |  |
| A5 | Does outdoor gardening during day time. |  |  |  |
| A6 | Seek protection of shady areas when I am outdoor. |  |  |  |
| A7 | Go to shopping centre to cool down. |  |  |  |
| A8 | Listen to daily weather forecast. |  |  |  |
| A9 | Use umbrella when walking outside. |  |  |  |
| A10 | Buy air conditioning. |  |  |  |
| A11 | Buy fan. |  |  |  |
| A12 | Live in more shady places(indoor/outdoor) |  |  |  |
| A13 | Make additional green/blue around house. |  |  |  |
| A14 | Use thin or no bedding. |  |  |  |
| A15 | Wear thin or no clothes while sleeping at night. |  |  |  |
| A16 | Put wet clothes in open window. |  |  |  |
| A17 | Keep windows open during night time. |  |  |  |
| A18 | Let air in room before going to bed. |  |  |  |
