# Supplementary material for: Development, validation and reliability of scales and items for heat wave risk assessment of pregnant women
Source: Int J Biometeorol. 2024 Aug 29;68(11):2205–14. doi: 10.1007/s00484-024-02738-x (PMC11519304; doi:10.1007/s00484-024-02738-x)
Supplement: Supplementary file 4 — Supplementary table 1- 5 [file 484_2024_2738_MOESM4_ESM.docx]

**Supplementary Tables**

**Supplementary table 2: KMO and Bartletts test of Specificity**

| **KMO Value** | 0.58 |
| --- | --- |
| **Bartlett’s test of specificity**  **Significant** | 0.00 |

**Supplementary Table 3: Communalities of the initial solution**

| **Items** | **Initial** | **Extraction** |
| --- | --- | --- |
| K1 | 0.46 | 0.40 |
| K2 | 0.55 | 0.45 |
| K3 | 0.50 | 0.51 |
| K4 | 0.64 | 0.73 |
| K5 | 0.42 | 0.38 |
| K6 | 0.63 | 0.66 |
| K7 | 0.51 | 0.55 |
| K8 | 0.39 | 0.43 |
| PV1 | 0.86 | 0.86 |
| PV2 | 0.78 | 0.79 |
| PV3 | 0.69 | 0.67 |
| PV4 | 0.85 | 0.85 |
| PS1 | 0.71 | 0.69 |
| PS2 | 0.75 | 0.68 |
| PS3 | 0.75 | 0.79 |
| PS4 | 0.55 | 0.60 |
| PS5 | 0.56 | 0.50 |
| PB1 | 0.39 | 0.23 |
| PB2 | 0.49 | 0.55 |
| PB3 | 0.45 | 0.52 |
| PB4 | 0.40 | 0.34 |
| PB5 | 0.58 | 0.66 |
| PB6 | 0.48 | 0.80 |
| Pba1 | 0.49 | 0.56 |
| Pba2 | 0.63 | 0.86 |
| Pba3 | 0.48 | 0.46 |
| Pba4 | 0.55 | 0.52 |
| Cu1 | 0.74 | 0.63 |
| Cu2 | 0.73 | 0.71 |
| Cu3 | 0.81 | 0.90 |
| Cu4 | 0.80 | 0.75 |
| Cu5 | 0.48 | 0.91 |
| A1 | 0.50 | 0.49 |
| A2 | 0.35 | 0.26 |
| A3 | 0.41 | 0.42 |
| A4 | 0.38 | 0.47 |
| A5 | 0.41 | 0.43 |
| A6 | 0.46 | 0.38 |
| A7 | 0.48 | 0.41 |
| A8 | 0.58 | 0.62 |
| A9 | 0.45 | 0.46 |
| A10 | 0.37 | 0.35 |
| A11 | 0.43 | 0.35 |
| A12 | 0.46 | 0.40 |
| A13 | 0.41 | 0.38 |
| A14 | 0.41 | 0.31 |
| A15 | 0.49 | 0.37 |
| A16 | 0.41 | 0.36 |
| A17 | 0.62 | 0.64 |
| A18 | 0.59 | 0.57 |

**Supplementary tables 4.1 Correlation Matrix of Factor 1**

| **PV3** | **PV3** | **PS1** | **PS2** | **PS3** | **PS5** |
| --- | --- | --- | --- | --- | --- |
|  | 1.0 |  |  |  |  |
| **PS1** | 0.42 | 1.0 |  |  |  |
| **PS2** | 0.52 | 0.57 | 1.0 |  |  |
| **PS3** | 0.51 | 0.68 | 0.64 | 1.0 |  |
| **PS5** | 0.48 | 0.31 | 0.41 | 0.41 | 1.0 |
| Average: 0.66 | | | | | |

**Supplementary tables 4.2. Correlation Matrix of Factor 2**

| **Cu1** | **Cu1** | **Cu2** | **Cu3** | **Cu4** |
| --- | --- | --- | --- | --- |
|  | 1.0 |  |  |  |
| **Cu2** | 0.58 | 1.0 |  |  |
| **Cu3** | 0.52 | 0.74 | 1.0 |  |
| **Cu4** | 0.70 | 0.57 | 0.69 | 1.0 |
| Average: 0.78 | | | | |

**Supplementary tables 4.3 Correlation Matrix of Factor 3**

| **PV1** | **PV1** | **PV2** | **Pv4** |
| --- | --- | --- | --- |
|  | 1.0 |  |  |
| **PV2** | 0.79 | 1.0 |  |
| **PV3** | 0.77 | 0.69 | 1.0 |
| Average: 0.87 | | | |

**Supplementary tables 4.4. Correlation within the factor**

| **Factor** | **1** | **2** | **3** | **Overall** |
| --- | --- | --- | --- | --- |
| **Average within factor correlation** | 0.66 | 0.78 | 0.87 | 0.77 |

**Supplementary table 5. KMO value of the final solution**

| **Kaiser –Meyer-Olkin (Measure of Sampling Adequacy)** | 0.80 |
| --- | --- |
